# Nephrotic range proteinuria in c-ANCA-positive crescentic glomerulonephritis with linear immune deposits

**DOI:** 10.4103/0971-4065.45294

**Published:** 2008-10

**Authors:** N. P. Singh, S. Gulati, V. Garg, P. Beniwal, S. Garg

**Affiliations:** Department of Medicine, Maulana Azad Medical College, New Delhi-110 002, India

**Keywords:** Anti-GBM disease, nephrotic syndrome, rapidly progressive glomerulonephritis

## Abstract

The three broad groups of rapidly progressing glomerulonephritis are anti glomerular basement membrane (anti-GBM) disease, renal vasculitis characterized by antineutrophil cytoplasmic antibody positivity, and a heterogeneous group with granular immune deposits. Anti-GBM disease with cytoplasmic antineutrophilic antibodies (c-ANCA) positivity (type III disease) is not known to present with nephrotic syndrome. We report here a rare presentation of nephrotic syndrome in Type III disease. Larger studies are warranted to determine whether the amount and/or type of immune deposits decide the range of proteinuria. These studies are also required to elucidate the impact of immune complex deposition on renal disease in c-ANCA-positive glomerulonephritis and to outline its pathogenetic mechanism.

## Introduction

Rapidly progressing glomerulonephritis (RPGN) is a disease of the kidney that results in a rapid decrease in the glomerular filtration rate by at least 50% over a short period (a few days to three months). The main pathological finding is fibrinoid necrosis (> 90% of biopsy specimens) and extensive crescent formation is present in at least 50% of the glomeruli. Three broad groups of crescentic glomerulonephritis can be distinguished, namely, anti-GBM disease which has circulating antibodies against glomerular basement membrane and linear deposits of the antibody along the membrane, renal microscopic vasculitis associated with ANCA positivity, and a heterogeneous group which has granular immune deposits and proliferative glomerulonephritis complicated by crescent formation. c-ANCA-positive crescentic glomerulonephritis with or without anti-GBM disease, is usually associated with scanty deposits of immunoglobulins and a nonnephrotic range of proteinuria. We report here a case of crescentic glomerulonephritis with c-ANCA positivity (Type III disease) and significant linear immune deposits and an unexpected nephrotic range of proteinuria.

## Case Report

A 51 year-old heavily built male patient presented to the hospital with a history of gradually progressing anasarca of two months’ duration. Initially, the patient had had pedal edema which progressed later on to involve the whole body. There were no significant cardiorespiratory complaints. On screening, the patient was found to have significant proteinuria and was referred to us.

The patient had a significant medical history: he was a known hypertensive for seven years on regular treatment, had dyslipidemia, was diagnosed as having obstructive sleep apnea, and had a history of significant NSAID intake five years ago for around one year for vascular headaches. His medications included ramipril, losartan potassium, and atorvastatin. Obstructive sleep apnea was being managed conservatively. There was a positive family history of hypertension. Of significance is the fact that he denied any recent history of NSAID intake.

Physical examination found a 97 kg, afebrile man with a BMI of 32.01 kg/m^2^ and a blood pressure of 140/90 mm Hg. He had mild pedal edema but the results of the other aspects of the general physical and systemic examination was normal. Laboratory results included a hemoglobin level of 15.4 g/dL (154 g/L), white blood cell count of 10,800 × 10^3^/µL (×10^9^/L), ESR of 55 mm, blood urea of 50 mg/dL, serum creatinine of 2.4 mg/dL, estimated CCR of 50 ml/min, total cholesterol of 198 mg/dL, serum triglyceride level of 151 mg/dL, serum HDL cholesterol level of 42 mg/dL (on statin therapy), total serum protein level of 6.2 g/dL, and a serum albumin level of 2.8 g/dL. Urine was suggestive of 4+ proteinuria and the 24-h urinary protein level was 4.9 g/24 h and was positive for active sediments. The results of thyroid function tests were within the normal range and echocardiography was suggestive of concentric left ventricular hypertrophy with type 1 diastolic dysfunction. Ultrasonography of the abdomen was suggestive of hepatomegaly with grade 2 fatty liver and a right kidney with a size of 12.5 cm × 6.3 cm and a left kidney of size 13 cm × 6.4 cm. Both the kidneys had normal echogenicity and had maintained their corticomedullary differentiation. Some of the special investigations have been shown in Table 1.

**Table 1:** Special investigations

Investigations	Result
HIV 1 and 2	Negative
Hepatitis B surface antigen	Negative
Antihepatitis C antibodies	Negative
Antistreptolysin O titers	Negative
Antinuclear antibodies	Negative
Serum complement C3	Normal
Serum complement C4	Normal
Cytoplasmic antineutrophilic antibodies (c-ANCA)	Positive
Perinuclear antineutrophilic antibodies (p-ANCA)	Negative
Antiglomerular basement membrane antibodies (antiGBM)	Negative

**Fig. 1:**
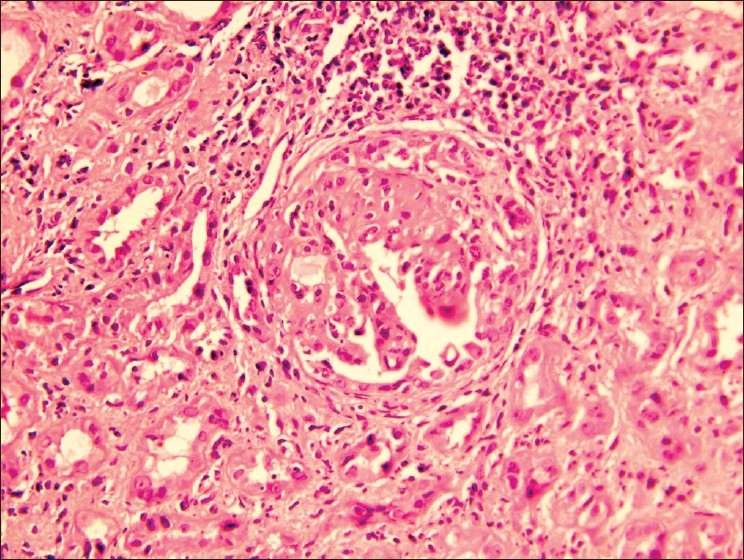
A glomerulus showing fibrocellular crescents (H&E ×150)

**Fig. 2:**
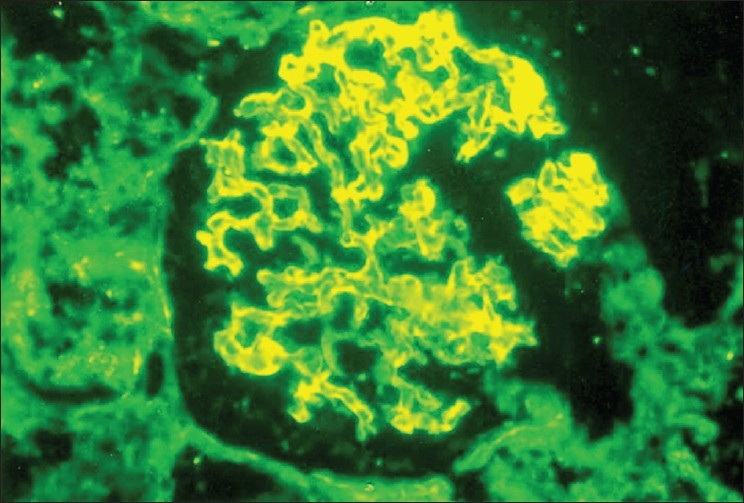
Immunofluorescence staining showing glomeruli with linear positivity for IgG (×400)

Kidney biopsy showed ten glomeruli, five of which showed partial to complete fibrocellular crescents [Fig. 1]. The capillary basement membrane was thickened and wrinkled and there was a focal increase in the mesangial matrix and cellularity; no spikes could be seen. There was marked tubular atrophy, interstitial edema, and fibrosis as well as a diffuse mononuclear cell infiltrate in the interstitium. The vessels and the basement membrane were unremarkable. On immunofluorescence, glomeruli showed linear positivity for IgG (++), IgM (+), fibrinogen (+), and focal IgM and IgA deposition [Fig. 2]. The diagnosis was crescentic glomerulonephritis with significant immune deposits.

The patient was started on intravenous, pulsed methyl prednisolone therapy along with oral prednisolone (1 mg/kg). The renal functions of the patient improved for a while, only to worsen within 2–3 months. Our patient also developed side effects of corticosteroid therapy. At this point, intravenous, pulsed cyclophosphamide was started. Despite all our efforts, his renal functions continued to deteriorate and he was put on maintenance hemodialysis.

## Discussion

Rapidly progressing glomerulonephritis can be due to anti-GBM disease, immune complex-associated disease, or crescentic glomerulonephritis with c-ANCA positivity. c-ANCA-positive, crescentic glomerulonephritis is usually pauciimmune and is not associated with nephrotic-range proteinuria. Type III disease (anti-GBM + ANCA) is also not known to have nephrotic range proteinuria. This raises question, whether the amount and/or type of immune deposits in c-ANCA-positive, crescentic glomerulonephritis decide the range of proteinuria?

There was linear positivity for immunoglobulins, which is very characteristic of antiglomerular basement membrane (anti-GBM) disease. Direct immunofluorescence of the renal biopsy is the most sensitive technique of all for anti-GBM disease, provided adequate renal tissue can be obtained and glomerular destruction is not severe. However, there may be occasional false-positive results as linear fluorescence has been noted in a number of circumstances other than Goodpasture's disease. Alport's syndrome after renal transplantation, systemic lupus erythromatosis, Diabetes Mellitus, normal autopsy kidneys, cadaver kidneys after perfusion, transplant biopsies, and fibrillary nephritis all lead to linear fluorescence of the renal biopsy. The anti-GBM antibody titers may be relatively lower in c-ANCA- and anti-GBM-positive patients.^1^,^2^ In this light, concomitant anti-GBM disease, even with negative anti-GBM titers cannot be ruled out in our patient, making combined c-ANCA and anti-GBM disease (*i.e*., Type III disease) a strong possibility.

Mixed essential cryoglobulinemia is usually associated with membranoproliferative glomerulonephritis, but occasionally, it may be associated with severe crescentic disease. Thus, testing for cryoglobulins was necessary, but could not be done because test was not available in our institute.

Neumann *et al.* published a report suggesting increased proteinuria in patients with crescentic glomerulonephritis associated with glomerular immune deposits.^3^ In this report, out of 45 patients with a recent onset of Wegner's granulomatosis, microscopic polyangiitis, or idiopathic crescentic glomerulonephritis, eight (18%) patients had significant proteinuria and significant immune deposits. This group was associated with worse prognosis. Our patient had nephrotic-range proteinuria along with linear positivity for IgG (++), IgM (+), fibrinogen (+), and focal IgM and IgA deposition in the glomeruli on immunofluorescence staining and c-ANCA positivity. Our report is one of the few case reports highlighting increased proteinuria with increased amount of linear immune deposits and increased c-ANCA positivity (Type III disease). We could find only a handful of case reports with similar findings which have been shown in Table 2.

**Table 2:** Case reports of c-ANCA positivity with immune complex deposition

Author/Year	No. of cases	ANCA	Renal histology (location)
Andrassy *et al*.^4^	4	c-ANCA	IgA, C3 (mesangial, peripheral)
Rollino *et al*.	1	c-ANCA	IgA, C3 (mesangial, GBM)
Hass *et al*.^5^	6	3 c-ANCA, 1 p-ANCA, 2 MPO +	IgM, IgA, C3 (mesangial)
Aasarod *et al*.	2	c-ANCA	IgM, IgA (mesangial)
Neumann *et al*.^3^	8	5 c-ANCA, 3 p-ANCA	IgA, IgG, IgM, C3 (mesangial, sub endothelial)
Our report	1	c-ANCA	IgG, IgM, IgA (glomeruli)

Neumann *et al.* hypothesized that immune deposits are found in the early part of crescentic glomerulonephritis in animal models and that they decrease with the passage of time. The kidney biopsy is a static record of the dynamic process of crescentic glomerulonephritis; it still might be possible that the immune deposits were present at an earlier time and had been decreased by phagocytosis and digestion by infiltrating neutrophils.^3^ It has been noted that the presence of ANCA aggravates and hastens the glomerular disease. Similarly, ANCA might also hasten the damage done by immune complexes and lead to increased proteinuria.^3^

Obesity, hypertension, dyslipidemia, NSAID intake, and obstructive sleep apnea all are independent risk factors for renal disease and might have been a few confounding variables in our case. Apart from the well recognized association with obesity of hypertension and diabetes mellitus, proteinuria is common in patients attending clinics for the treatment of morbid obesity. Metcalf *et al*. noted a strong relationship between subclinical levels of proteinuria and the body mass index in a population study of nearly 6000 subjects aged more than 45 years.^6^ Proteinuria in obese patients may be sufficient to induce nephrotic syndrome and it may diminish or disappear with weight loss. Renal histology has been studied in only a few obese patients with nephrotic syndrome and ranges from minimal change disease and membranous nephropathy with renal vein thrombosis, to focal segmental glomerulosclerosis. Our patient was obese with a BMI of 32.01 kg/m^2^, but apart from having nephrotic range proteinuria, he also had active urinary sediments and c-ANCA positivity. Active urinary sediment, c-ANCA positivity, and crescentic glomerulonephritis with significant linear immune deposits on renal biopsy point towards renal involvement unrelated to obesity.

In dyslipidemia, lipids may directly damage previously injured glomerular and tubular structures. Correcting dyslipidemias may help slow the rate of the functional decline in patients with progressive renal disease. Nephrotic syndrome may lead to dyslipidemias but not *vice versa*. Nephrotic syndrome in our patient could not be attributed to the dyslipidemic state diagnosed before the initiation of the renal disease.

NSAIDs are potentially nephrotoxic and their effects include salt and water retention, acute tubular necrosis, acute interstitial nephritis with heavy proteinuria, hyperkalemia, and chronic renal failure. There are anecdotal reports of generalized vasculitis and glomerulonephritis in patients taking NSAIDs. Other than minimal-change nephropathy being associated with an acute interstitial nephritis, the evidence that NSAIDs lead to glomerulonephritis is unconvincing. Acute allergic tubulointerstitial nephritis due to NSAIDs is much less common than the hemodynamic form of renal failure. The patients are often elderly and the drug may have been taken for months or years before the development of acute interstitial nephritis. There is often little clinical evidence of an allergic reaction. The nephrotic range of proteinuria in NSAID-induced tubulointerstitial nephritis is an unusual feature. This is a particular feature of fenoprofen-induced tubulointerstitial nephritis.^7^ The insidious nature of the onset of NSAID-induced tubulointerstitial nephritis and the wide use of NSAIDs make it important to carefully obtain a drug history in patients with unexplained renal failure. Urinary active sediments, crescentic glomerulonephritis associated with significant linear immune complex deposits, and c-ANCA positivity rule out NSAIDs as the primary etiological agent.

Some initial studies had suggested an association between obstructive sleep apnea and proteinuria. Several factors including hypertension, hypoxemia, hyperlipidemia, and increased sympathetic nerve activity contribute to a progressive decline in renal function. Two recent studies highlighted the fact that proteinuria in patients of obstructive sleep apnea should not be attributed to sleep apnea, a fact that warrants further evaluation.^8^–^9^ A careful literature search did not reveal any study on renal histological findings in patients of obstructive sleep apnea. Active urinary sediments, c-ANCA positivity, and the renal biopsy findings like those in our patient are not seen in this group of patients with sleep apnea.

To summarize, a 51 year-old obese male with a history of hypertension and obstructive sleep apnea, who had dyslipidemia and a history of NSAID intake, presented with nephrotic syndrome. Renal biopsy showed crescentic glomerulonephritis along with linear immune deposits characteristic of anti-GBM disease. c-ANCA positivity along with the linear immune deposits in renal biopsy supports the possibility of Type III disease (c-ANCA + anti-GBM) in our patient. Whether the immune deposits constituted the sole cause of increased proteinuria or whether many confounding variables were at play along with crescentic glomerulonephritis is a perplexing issue. To the best of our knowledge, this is a rare case report of nephrotic syndrome in Type III disease. Larger studies are warranted to elucidate the impact of immune complex deposition on renal disease in c-ANCA-positive glomerulonephritis and to outline its pathogenetic mechanism.
